# Nano-Modified Vibrocentrifuged Concrete with Granulated Blast Slag: The Relationship between Mechanical Properties and Micro-Structural Analysis

**DOI:** 10.3390/ma15124254

**Published:** 2022-06-15

**Authors:** Alexey N. Beskopylny, Evgenii M. Shcherban’, Sergey A. Stel’makh, Levon R. Mailyan, Besarion Meskhi, Alexandr Evtushenko, Valery Varavka, Nikita Beskopylny

**Affiliations:** 1 Department of Transport Systems, Don State Technical University, Gagarin Sq. 1, 344003 Rostov-on-Don, Russia; 2 Department of Engineering Geology, Bases and Foundations, Don State Technical University, Gagarin Sq. 1, 344003 Rostov-on-Don, Russia; au-geen@mail.ru (E.M.S.); sergej.stelmax@mail.ru (S.A.S.); 3 Department of Roads, Don State Technical University, Gagarin Sq. 1, 344003 Rostov-on-Don, Russia; lrm@aaanet.ru; 4 Department of Life Safety and Environmental Protection, Faculty of Life Safety and Environmental Engineering, Don State Technical University, Gagarin Sq. 1, 344003 Rostov-on-Don, Russia; spu-02@donstu.ru; 5 Department of Unique Buildings and Constructions Engineering, Don State Technical University, Gagarin Sq. 1, 344003 Rostov-on-Don, Russia; a.evtushenko@mail.ru; 6 Research and Education Center “Materials”, Don State Technical University, Gagarin Sq. 1, 344003 Rostov-on-Don, Russia; varavkavn@gmail.com; 7 Department of Hardware and Software Engineering, Don State Technical University, Gagarin Sq. 1, 344003 Rostov-on-Don, Russia; beskna@yandex.ru

**Keywords:** vibrocentrifuged concrete, nano-modified concrete, variotropic structure, ground granulated blast-furnace slag

## Abstract

Currently, in civil engineering, the relevant direction is to minimize the cost of the manufacture of the hollow structures of annular sections, as well as their construction and installation efficiency. To optimize the costs associated with building products and structures, it is proposed to apply the technology of vibrocentrifugation, to reconsider and comprehensively approach the raw materials for the manufacture of such products and structures. The purpose of this study is a theoretical substantiation and experimental verification with analytical numerical confirmation of the possibility of creating improved variotropic structures of vibrocentrifuged concrete nano-modified with ground granulated blast-furnace slag. The study used the methods of electron microscopy, laser granulometry, and X-ray diffraction. Slag activation was carried out in a planetary ball mill; samples were prepared on a special installation developed by the authors—a vibrocentrifuge. The optimal and effective prescription–technological factors were experimentally derived and confirmed at the microlevel using structural analysis. The mathematical dependencies among the composition, macrostructure, microstructure, and final properties of vibrocentrifuged concrete nano-modified by slag are determined. Empirical relationships were identified to express the variation of some mechanical parameters and identify the relationship between them and the composition of the mixture. The optimal dosage of slag was determined, which is 40%. Increases in strength indicators ranged from 16% to 27, density—3%.

## 1. Introduction

### 1.1. Background

Nowadays, the actual problem of current construction is the need for new building materials, structures, products, and technologies to obtain the most efficient buildings and structures in terms of ecology, energy saving, manufacturability, minimization of labor intensity, material consumption, resource intensity, and energy intensity. In this regard, the most reliable building structures, which contain small sections, are promising, allowing the construction industry to significantly reduce the weight of the resulting buildings and structures, which at the same time have high performance characteristics and are characterized by efficiency at all stages of the life cycle of construction products and construction processes.

**Figure 1:**
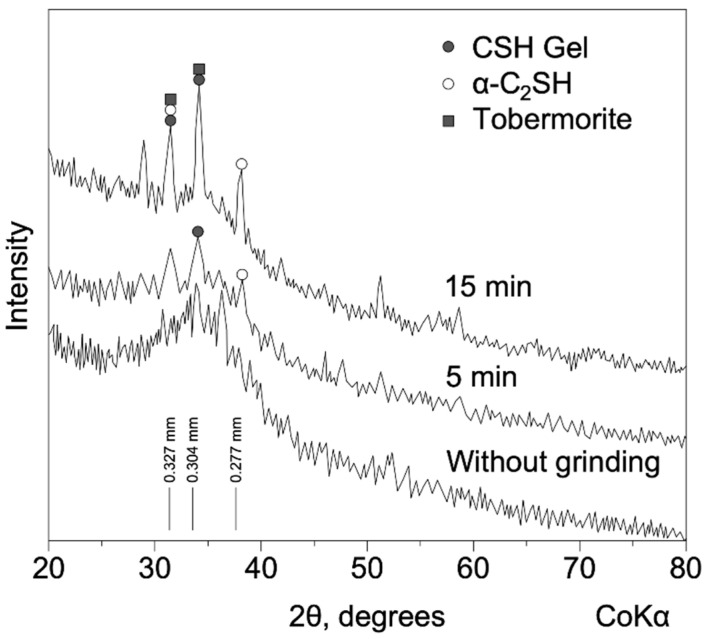
Diffractograms of hydrated slag at different grinding times after holding for 28 days (based on [3]).

The most important factor in the effective replacement of part of the cement composition with blast-furnace slag is the correct selection and grinding of the components. In [1,2,3], slag and clinker which were preliminarily ground in a friction mill were analyzed. It was established that the grinding time makes it possible to significantly reduce the particle size. However, from a specific moment, a high increase in the zeta-potential (the potential difference that exists between the surface of a solid particle immersed in a conducting liquid (e.g., water) and the bulk of the liquid) is noted, which characterizes the ability of the system to be in a colloidal state. In addition, the diffraction pattern in Figure 1 shows the phases formed during the hydration of slag of various dispersities. A higher dispersity makes it possible to obtain phases of hydrated compounds with more pronounced peaks in the final material [3,4].

This study of the strength properties of the prototypes showed that it is possible to replace up to 85% of clinker with slag, while the final material demonstrates indicators that are not inferior to concrete on clinker binders. The most significant drawback of the samples is the lack of the “required strength in the early stages of hardening” [1,2,3,4].

An analysis of the effect of slag dispersion on the manifestation of hydraulic activity when added to cement was carried out in [5]. A ball mill was used to grind the slag, the grinding times were 1, 3, 6, and 9 h. The amount of slag in the final samples was 45%. In the study’s course, it was found that during the grinding process it is possible to achieve a specific surface area of 1.8 m^2^/g. At this value, the highest tensile strength values were observed [4,5].

In [6], a mechanical method for activating a slag solution from ordinary Portland cement (PC) was researched. “The first group includes both ground slag and ordinary PC. In the second group, ground slag and unground slag were used, and in the third group, ground slag and unground slag were used. All three groups of mortars used showed a significant effect on the increase in strength, especially at an early age” [6]; this was in contrast to [1,2,3]. The results obtained proved that the loss of strength at a later age occurs due to “phase separation”. “It was found that ground slag has a greater effect on the increase in strength compared to ground PC particles. Fine grinding results in a larger surface area, but agglomeration is a limitation of the specific surface area.” [6]

The issue of studying the influence of the specific surface area of ground granulated slag was also studied in [7,8,9,10,11]. The crucial result of the conducted research is the identification of an extremum on the curve of dependence of the strength of the final material on the specific surface area. It has been established that there is a threshold value that corresponds to the highest strength index when replacing part of the cement with ground blast-furnace slag (GBFS). The authors explained this effect through the aggregation of small particles due to the appearance of electrostatic forces of attraction [4,7,8,9].

In many works [12,13,14,15,16,17,18,19,20,21,22,23,24,25,26,27,28,29,30,31,32] on the study of the possibility of using slags as raw materials for building materials, the importance of the chemical and phase compositions of materials is noted; this largely determines the possibility of the hydration process and strength development, as well as the type of alkaline component used in mixing and the conditions of the hardening environment [4].

The effect of the hardening temperature on the concrete properties with the addition of ground granulated blast-furnace slag (GGBFS) was investigated in [17,28,30], in which an analysis of the dependence of properties on the formulations and hardening temperatures of concrete was conducted. In [17], an analysis is undertaken of the influence of the hardening temperature on the final strength of artificial stones consisting of 50% slag, 50% fly ash, and the alkaline additive NaOH (10 M). The samples were hardened at temperatures of 22 and 65 °C. The results are presented in Table 1.

Thus, during the study, it was found that an increase in the hydration temperature does not have an important effect on the “strength properties of the samples” [4,17].

The work [21] presents “the results of a study of the effect of sodium hydroxide, sodium carbonate and sodium silicate as activators and temperatures in the range of 20–31 °C on the setting time and workability of freshly activated slag-cement (ASC) mixtures”. Following the results of experimental studies, it was determined that slag–cement mixtures have a shorter setting time than a mixture of ordinary Portland cement. The maximum setting acceleration was recorded “with sodium silicate, while sodium hydroxide and sodium carbonate show a moderate effect. As for the temperature, its increase is accompanied by an acceleration of setting and a decrease in the mixture’s workability” [21].

The issue of the durability of concretes and mortars based on slag has also received much attention [26,30,32,33,34,35,36]. At the same time, these characteristics of solutions with the addition of DHS as frost resistance [30] and resistance to chlorides [34], sulfates [33], and various acids [26,32] were studied. Moreover, the authors of many works consider the joint effect of GBFS and other additives (ash, limestone, gypsum, nanosilica) on the formation of the micro- and macrostructure of materials based on them and their physical and mechanical characteristics [10,28,30,35,36,37,38].

Special focus should also be paid to the combined effect of slag and other dispersed additives on the properties of composites. Fly ash, nanosilica, lime, and gypsum, together with slag, improve the individual properties of composites based on them [39,40,41,42].

### 1.2. Rationale

Thus, our goal is a comprehensive development aimed at the enhancement of the characteristics of the building materials used in obtaining effective building structures of buildings and structures. One of the most effective types of building structure is the hollow structures with an annular section, primarily obtained via centrifugal compaction methods [43]. This method, in turn, as is known from our previous works and the works of other authors, can be improved by transforming it using the vibrocentrifugation method [43,44,45,46,47,48]. This aims to achieve the so-called variotropic structure in such concrete and in such structures. Variotropic structure is a term first introduced by the authors in previous studies, which means a structure, heterogeneous in cross-section, of centrifugally compacted cement concrete with different physical and mechanical characteristics along the thickness of the annular section. This structure is perfect in products and structures with an annular section and allows the most efficient use of available reserves, as well as hidden resources in new-generation reinforced concrete structures.

A relevant research direction is also minimizing the cost of manufacture of these products and their construction and installation efficiency. To optimize the cost of building products and structures, first, it is necessary to review and comprehensively approach the raw materials, as well as the technology for manufacturing such products and structures. If, from the point of view of technology, we can already talk about the effectiveness of using the vibrocentrifugation method instead of simple centrifugation, then the question regarding building materials is still open.

In this regard, the working hypothesis of the study is the development of the most cost-effective formulation with the maximum use of cheap raw materials, which can primarily be obtained from industrial and other types of waste used for manufacturing such products. One of the most demanded is the use of the production waste of the fuel and energy complex, i.e., production waste obtained during the operation of industrial and agro-industrial facilities, which is discussed in [1,2,3,4,5,6,7,8,9,10,11,12,13,14,15,16,17,18,19,20,21,22,23,24,25,26,27,28,29,30,31,32,33,34,35,36,37,38,39,40,41,42,49,50,51,52,53,54,55,56].

In the present work, the influence of such waste as GBFS on the possibility of obtaining the most effective variotropic structure of products and structures with an annular section obtained through centrifugal compaction methods is studied. According to the stages of work, the most important aspects should be noted, such as the prescription (that is, the qualitative and quantitative characteristics of the raw materials used) and the technological (i.e., the relationship between the modes of centrifugal compaction and the characteristics of the resulting variotropic improved concrete based on waste in the form of blast-furnace granulated slag). the evaluation of the structural efficiency of such a method using prescription and technological methods due to mathematical, statistical, and resulting processing and other analytical tools to determine the most desirable modes and recipes is necessary.

And, finally, the constructive aspect, expressed in terms of the construction and installation efficiency and practical applicability of the developed approaches is discussed. At the same time, the structural aspect should be considered not only at the macro level during the formation of coarse variotropic structures, but also from the point of view of microscopic examination to understand the fundamental processes that occur during the interaction of raw materials, both with the use of industrial waste and with more standard typical components and raw materials.

The purpose of this research is the study of the combination of vibrocentrifugation technology and the nano-modification of concrete with blast-furnace granulated slag, i.e., the theoretical and experimental verification of the creation of variotropic nano-modified structures.

The novelty of the study is as follows.

The optimal recipe-technological parameters of the process of nano-modification by blast-furnace granulated slag of vibrocentrifuged concrete have been determined.

Microscopic examination has proven the improvement of the microstructure and macrostructure of new concrete by achieving a denser packing of particles in the working layers of a variotropic section.

New theoretical and experimental dependences of the structure and properties of vibrocentrifuged concretes on the parameters of its nano-modification have been obtained.

The scientific novelty of the study is the theoretically substantiated and experimentally and analytically confirmed possibility of obtaining a new, improved variotropic concrete with an improved structure at the macro- and microlevels due to the complex effect of nanomodification with granulated blast-furnace slag and optimal vibrocentrifuging compaction.

The research question underlying this article is the creation of a new type of vibrocentrifuged concrete with an improved variotropic structure obtained by its nanomodification with granulated blast-furnace slag. The subject of the study is the very possibility of obtaining such a concrete, as well as the choice of the rational formulation and technological parameters for its production.

**Figure 2:**
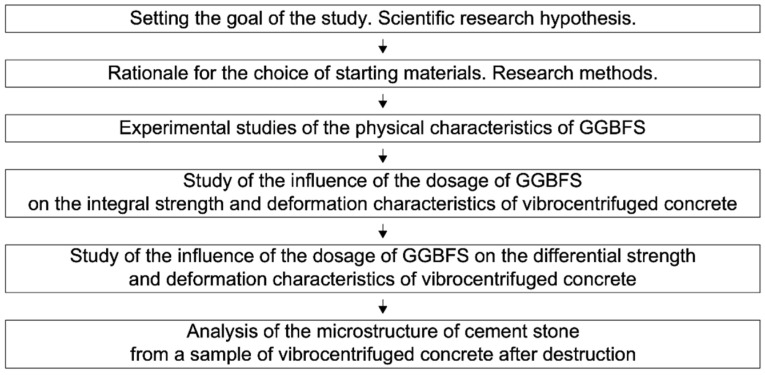
Program of experimental studies.

The scheme of this experimental study is shown in Figure 2.

## 2. Materials and Methods

### 2.1. Materials

Portland cement grade PC 500 D0 produced by OAO Novoroscement (Novorossiysk, Russia) was used as a binder in this study. The main Portland cement properties are as follows: specific surface—330 m^2^/kg, cement paste normal density—25%, fineness of grinding, passage through a sieve No. 008—95.7%, setting time—165–230 min, tensile strength in bending—4.6 MPa (after 2 days) and 7.6 MPa (after 28 days), and compressive strength—24.5 MPa (after 2 days) and 55.2 MPa (after 28 days). The mineralogical composition of Portland cement is presented in Table 2.

Quartz sand manufactured by OAO Arkhipovsky Quarry (Arkhipovskoe, Russia) was used as a fine aggregate. The physical characteristics of the sand are as follows: bulk density—1578 kg/m^3^, true density—2675 kg/m^3^, and content of dust and clay particles—1.1%, clay content in lumps—0.15%, and it is free of organic and contaminants. Grain composition and modulus of the sand sizes are given in Table 3.

Granite crushed stone produced by Pavlovsknerud JSC (Pavlovsk, Russia) was used as a large dense aggregate. Table 4 shows the characteristics of the crushed stone.

Granulated blast-furnace slag (GBFS) produced by NLMK PJSC (Lipetsk, Russia) was used as a mineral additive. The chemical composition of the blast-furnace slag is presented in Table 5.

Sodium hydroxide, NaOH, manufactured by PJSC Khimprom (Novocheboksarsk, Russia) was used as a chemical additive. The amount of this additive used for all experimental compositions was 8% by weight of the binder components. During the experimental studies, the sodium hydroxide powder was first dissolved in a certain small volume of mixing water, and then the resulting solution was mixed with the entire volume of mixing water.

### 2.2. Methods

To study the dispersity and morphology of the samples, electron microscopy and laser granulometry were used. The modified hardened cement paste structure was studied using a ZEISS CrossBeam 340 (Carl Zeiss Microscopy GmbH (Factory), Jena, Germany). The granulometric composition of the activated slag was evaluated using a MicroSizer-201C laser particle analyzer (OOO VA Install, St. Petersburg, Russia).

The study of the phase composition of the GBFS was carried out using X-ray diffraction on the device “Difrey” (JSC “Scientific Instruments”, St. Petersburg, Russia). The experimental curves were obtained with chromium radiation at room temperature. The shooting parameters on the X-ray tube were: voltage—25 kW, current—4 mA.

Slag activation (grinding) was carried out in the Activator-4M planetary ball mill (Novosibirsk, Russia).

The components of the slag contribute differently to the hydration process, which is determined by the interaction of some compounds with other components of the system. The binder properties of the GBFS were evaluated to predict their use more accurately in the composition of the final material. The evaluation of the binding properties of slags is determined from several indicators, such as the basicity modulus, activity index, basicity factor, and quality factor.

The basicity modulus was calculated using Equation (1):

(1) $$M_{O}=\frac{\mathrm{CaO}+\mathrm{MgO}}{{\mathrm{SiO}}_{2}{+\mathrm{Al}}_{2}O_{3}}$$

When the value of $M_{O}>1$, the slag is considered basic, at $M_{O}<1$ it is acidic, and at $M_{O}=1$ it is neutral.

The activity index is used to compare the hydraulic properties of slags. With an increase in the activity index, the hydraulic properties of slags increase. The higher the activity index, the greater the rate of curing.

The calculation of the activity index was carried out according to Equation (2):

(2) $$M_{a}=\frac{{\mathrm{SiO}}_{2}}{{\mathrm{Al}}_{2}O_{3}}$$

The basicity coefficient shows the ratio of the amount of calcium oxide to silicon oxide.

The calculation of the basicity coefficient was carried out according to Equation (3):

(3) $$K_{o}=\frac{\mathrm{CaO}}{{\mathrm{SiO}}_{2}}$$

The quality factor is used to determine the grade of blast-furnace slag according to GOST 3476 “Blast-furnace and electro thermos phosphoric granulated slags for cement production. Specifications”. It is noted that the higher the index, the higher the hydraulic activity of the slag [4].

The calculation of the quality factor was carried out according to Equation (4):

(4) $$K_{k}=\frac{{\mathrm{CaO}+\mathrm{Al}}_{2}O_{3}+\mathrm{MgO}}{{\mathrm{SiO}}_{2}{+\mathrm{TiO}}_{2}}$$

**Figure 3:**
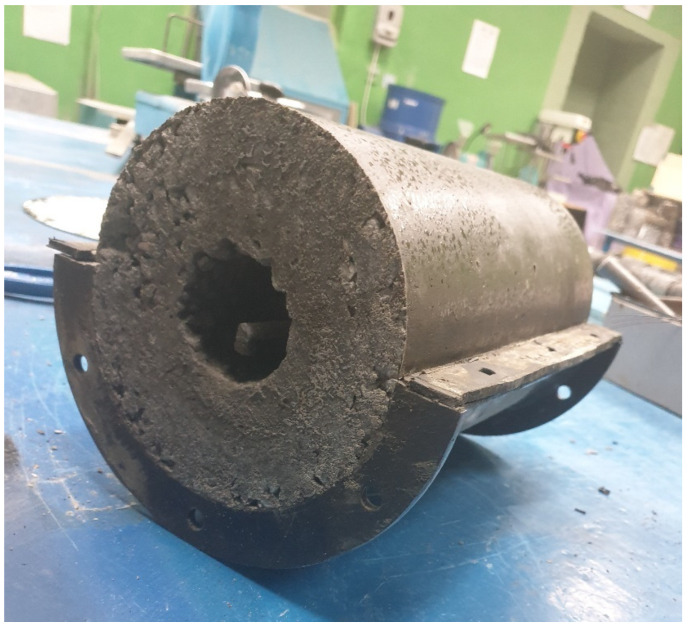
General view of the vibrocentrifuged prototype of the annular section.

For the manufacture of vibrocentrifuged samples, a special installation was used—a laboratory vibrocentrifuge [57]. The centrifugation parameters were: speed—156 rad/s and duration—12 min; vibration parameters: height of clamp projections—5 mm, projection length—20 mm, and pitch between projections—30 mm. The concrete-mix-forming parameters were taken from [45]. In general, the manufacturing technology of vibrocentrifuged prototypes includes the following main technological operations: dosing of concrete mix components, mixing concrete mix, determination of the characteristics of the workability of the resulting concrete mix, loading the concrete mixture into the mold, centrifugation process; sludge drain, exposure of the molded sample for 24 h in the form, and demolding of the prototype. The appearance of the obtained vibrocentrifuged prototypes is shown in the Figure 3.

The integral and differential characteristics of concrete were determined according to the method [44]. “Integral characteristics” are the overall characteristics, over the entire thickness of the section in an average form. The term “differential characteristics” means differences in values between different layers of the variotropic section and the determination of the characteristics of each layer separately to derive the calculated dependencies, taking into account the different characteristics of each layer. The program for manufacturing samples to determine the integral and differential indicators from a vibrocentrifuged prototypes of an annular cross section with dimensions (D = 450 mm is an outer diameter; d = 150 mm is an inner diameter; H = 1200 mm is the total height) is presented in [45].

For the manufacture and further testing of samples in this study, the same equipment and measuring instruments were used as in [45].

In total, nine vibrocentrifuged prototypes of annular cross-section were manufactured and tested. The dimensions of the cut samples were:

- to determine the integral characteristics—four cubes with an edge of 150 mm for compressive strength; one prism of 150 mm × 150 mm × 600 mm for the study of bending tensile strength; two prisms of 150 mm × 150 mm × 600 mm for axial compressive strength; and two prisms of 150 mm × 150 mm × 600 mm for the study of axial tensile strength; and
- to determine the differential characteristics—nine cubes with an edge of 50 mm for compressive strength; nine prisms of 50 mm × 50 mm × 200 mm for the study of bending tensile strength; nine prisms of 50 mm × 50 mm × 200 mm for axial compressive strength; and nine prisms of 50 mm × 50 mm × 200 mm for the study of axial tensile strength.

All samples were cut and tested in accordance with the requirements of GOST 28570 “Concretes. Methods of strength determination on cores selected from structures”. Detailed schemes for cutting samples for testing from vibrocentrifuged prototypes and a detailed description of the procedure for preparing samples for testing are presented in [44].

The strength of the manufactured samples, in terms of compression, tensile bending, and axial tension, was determined in accordance with the requirements of GOST 10180 “Concretes. Methods for strength determination using reference specimens”, and the axial compressive strength and modulus of elasticity were determined in accordance with the requirements of GOST 24452 “Concretes. Methods of prismatic, compressive strength, modulus of elasticity and Poisson’s ratio determination”. Measurements of the deformations of the concrete of the experimental prisms were carried out using a chain of strain gauges with a side length of 50 mm and dial indicators with a division value of 0.001 mm.

Moreover, an additional assessment was made of the variotropic efficiency of vibrocentrifuged concretes modified by the addition of slag. The values of the coefficients of variotropic efficiency were calculated according to the formulas given in Table 6.

For the manufacture of vibrocentrifuged samples with an annular section, heavy concrete of class B30 was designed in accordance with previously developed recommendations [44]. All concrete mixtures had the same workability grade (P1); the cone draft was 1–4 cm. Moreover, in the process of manufacturing experimental concrete mixtures, we were able to adjust the water flow to obtain the necessary rheological characteristics of the mixture. The content of fractions of coarse aggregate is represented by the following ratios: 60%—fraction 10–20 mm; 40%—fraction 5–10 mm. The parameters of the composition of the concrete mix obtained as a result of the calculations are shown in Table 7.

## 3. Results

### 3.1. Studies of the Physical Characteristics of the GBFS

Based on the data on the chemical composition of the GBFS, the calculation of indicators that determine the hydraulic properties of the presented material was carried out. The data obtained during the calculation are shown in Table 8.

**Figure 4:**
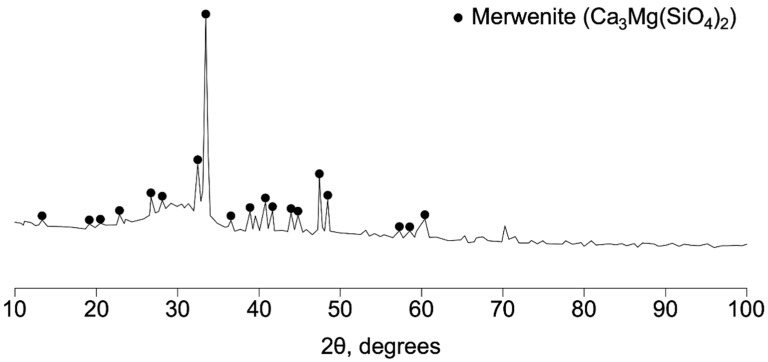
X-ray diffraction pattern of GBFS.

The amorphous phase (glass phase) is formed as a result of the content of a large amount of silicon in the samples and the method of cooling (granulation). The diffraction pattern obtained during the study of the DHS is shown in Figure 4.

On the diffraction curve of the GBFS sample (Figure 4), the intensity maxima of the mervenite phase (Ca_3_Mg(SiO_4_)_2_) are found; this phase is located in the middle of the sequence of hydraulic activity of substances present in blast-furnace slags and is characterized by low hydraulic properties. It can also be seen that this material contains a large number of amorphous phases. This theoretical justification is in good agreement with [4].

The data on the chemical composition of GBFS, as well as the conducted X-ray phase analysis, allow us to conclude that this GBFS can be used as an astringent mineral component.

Moreover, additional studies of GBFS were carried out in terms of indicators: moisture content, bulk and true density, and particle size distribution of crushed GBFS. The results of determining the physical properties of GBFS are as follows: humidity—1.9%, bulk density—1055 kg/m^3^, true density—2900 kg/m^3^.

To determine the optimal conditions for the activation (grinding) of GBFS, a number of experimental studies were carried out; to obtain samples of ground slag, grinding of GBFS was carried out at a speed of 400–500 rpm for 10, 20, 30, and 40 min.

**Figure 5:**
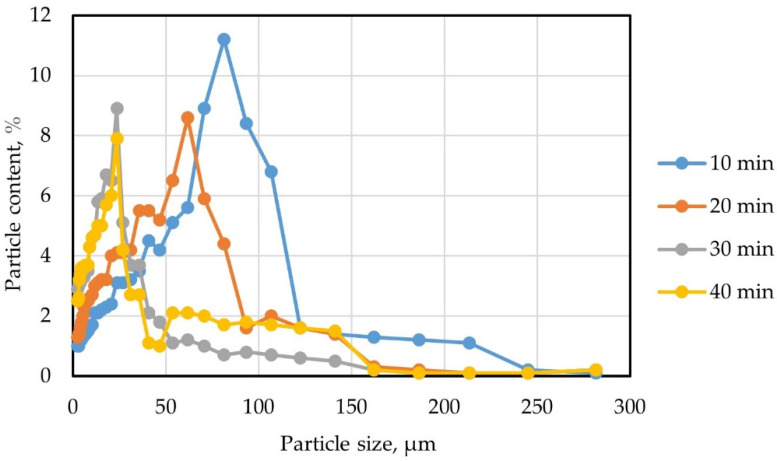
The distribution of GBFS particles by fractions from the grinding time in the Activator-4M planetary ball mill.

The particle size distributions of granulated slag after grinding for 10, 20, 30, and 40 min are shown in Figure 5.

It should be noted that, with an increase in the distribution time, significant changes in the size distribution of particles are observed. The observed effect can be explained by the fact that the Activator-4M ball mill, provided that the parameters and activation modes are rationalized, allows the equipment to be adjusted in such a way as to obtain a polydisperse particle distribution pattern. Such a picture, within certain limits, makes it possible to achieve a qualitative structure with the densest packing of particles; however, if rational activation ranges are exceeded, it can cause certain harm, due to the excessive grinding of functional particles. The influence of the grinding time of GBFS on its granulometric characteristics is reflected in Table 9.

Figure 5 and Table 9 illustrate the results of our own experimental studies, where, according to the results of granulometric analysis, it was found that grinding GBFS for 30 and 40 min makes it possible to obtain powders of a similar granulometric compositions with a predominant content of fractions in the size range of 50–150 microns. However, grinding for 30 min proved to be more effective, since, in that case, the maximum percentage of particles up to 50 microns in size was recorded.

According to [4], an important factor in determining the suitability of using slags as mineral binders is the fineness of material grinding. The fineness of grinding has a direct impact on the interfacial interaction “solid–liquid”, which causes the process of hydration to occur when interacting with water.

To determine the most desirable granulometric characteristics of the slag and the time of its grinding, additional studies were carried out. Four series of six sample-cubes with dimensions of 100 × 100 × 100 mm were made according to GOST 10180-2012 “Concretes. Methods for strength determination using reference specimens”. All prototypes were made from a concrete mixture with the same composition: one part of cement, three parts of sand, water–cement ratio 0.5, dosage of mineral additive in the amount of 15% replacement by weight of cement. The results of the tests of the sample-cubes for compressive strength are shown in Table 10.

Following the data of the results of the granulometric tests and the strength characteristics of the sample cubes, the most desirable granulometry contains GBFS, crushed for 30 min. Thus, in the future, for the implementation of the nano-modification of vibrocentrifuged concrete, a GBFS activated for 30 min was used.

### 3.2. The Effect of the GBFS Addition on the Physical, Mechanical, and Strain Characteristics of Vibrocentrifuged Concrete

During the experimental studies, part of the cement was replaced with activated slag in amounts of 10%, 20%, 30%, 40%, and 50%. The experimental results of the effect of the GBFS additive on the integral physical, mechanical, and strain characteristics of modified vibrocentrifuged concrete are presented in Table 11.

Table 11 shows that the values of the increase in strength and differential characteristics of vibrocentrifuged concretes modified by the addition of slag in amounts of 10, 20, 30, 40, and 50% compared with the control, amounted to:

- 7, 13, 14, 17, and 9%, respectively, for compressive strength;
- 5, 12, 12, 14, and 5%, respectively, for axial compressive strength;
- 6, 13, 15, 22, and 14%, respectively, for tensile strength in bending;
- 12, 16, 16, 20, and 10%, respectively, for axial tensile strength; and
- 6, 12, 13, 19, and 4%, respectively, for the modulus of elasticity.

**Figure 6:**
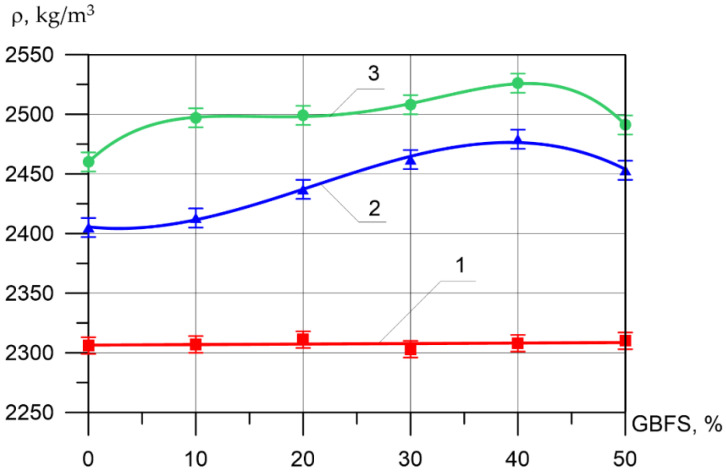
Dependence of the density of various layers of vibrocentrifuged concretes on the proportion of the modifying additive GBFS (here and in Figure 7, Figure 8, Figure 9, Figure 10 and Figure 11: 1—VC1 inner layer, 2—VC2 middle layer, 3—VC3 outer layer).

**Figure 7:**
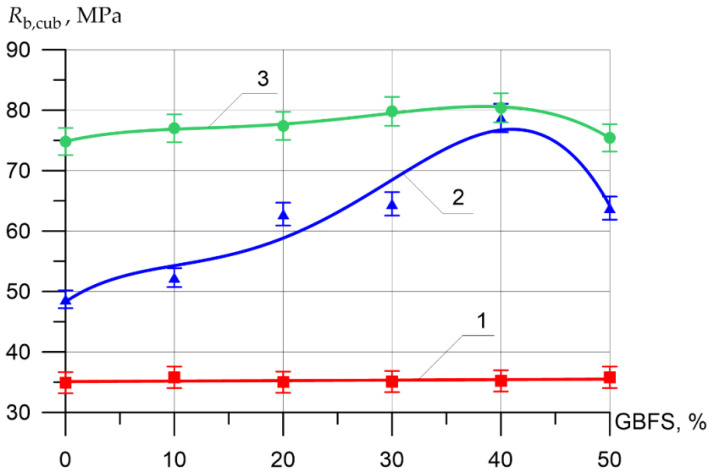
Compressive strength of various layers of vibrocentrifuged concretes related to the proportion of the modifying additive GBFS (1—VC1 inner layer, 2—VC2 middle layer, 3—VC3 outer layer).

**Figure 8:**
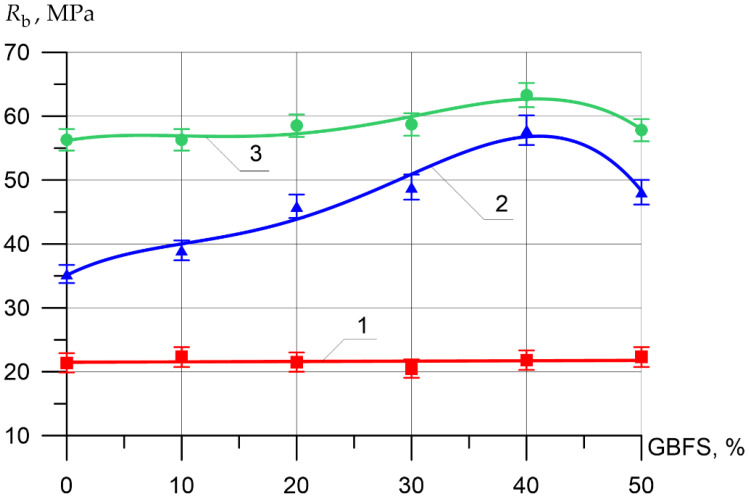
Axial compressive strength of various layers of vibrocentrifuged concretes related to the proportion of the modifying additive GBFS (1—VC1 inner layer, 2—VC2 middle layer, 3—VC3 outer layer).

**Figure 9:**
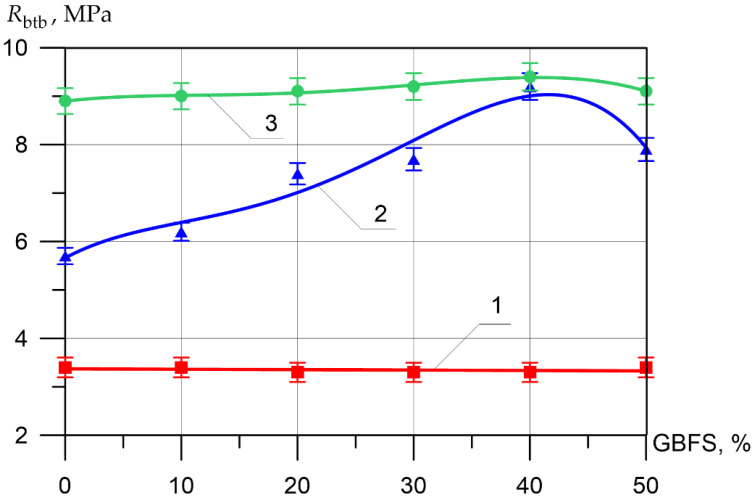
Tensile strength in bending of various layers of vibrocentrifuged concretes related to the proportion of the modifying additive GBFS (1—VC1 inner layer, 2—VC2 middle layer, 3—VC3 outer layer).

**Figure 10:**
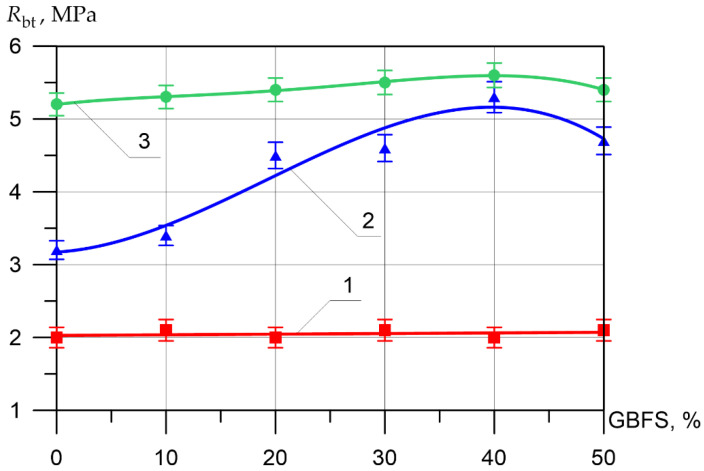
Axial tensile strength of various layers of vibrocentrifuged concretes related to the proportion of the modifying additive GBFS (1—VC1 inner layer, 2—VC2 middle layer, 3—VC3 outer layer).

**Figure 11:**
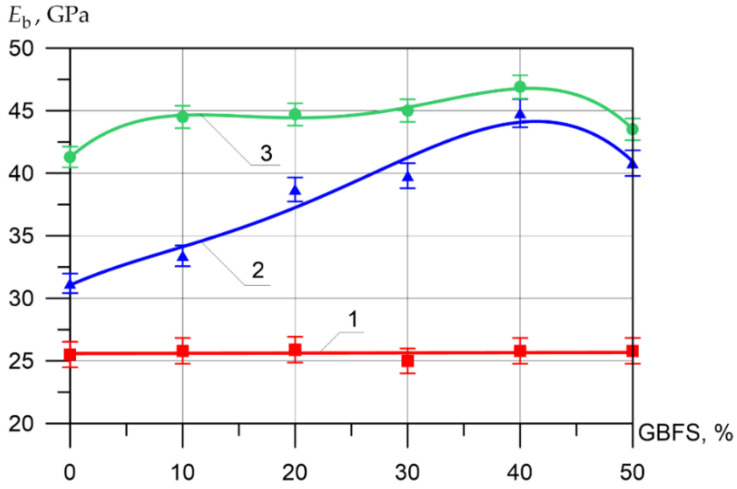
Modulus of elasticity of various layers of vibrocentrifuged concretes related to the proportion of the modifying additive GBFS (1—VC1 inner layer, 2—VC2 middle layer, 3—VC3 outer layer).

Changes in the differential physical–mechanical and deformative characteristics are shown in Figure 6, Figure 7, Figure 8, Figure 9, Figure 10 and Figure 11.

The outer layer of vibrocentrifuged concrete acquires a denser structure as a result of the action of the nano-modifier. The middle layer is also compacted simultaneously with hardening, while the inner layer remains, for example, at the same level due to the physical impact of centrifugal forces and the objective impossibility of controlling its characteristics with the help of nano-modification.

The mechanism of compaction and hardening with the help of a nano-modifier of the outer and middle layers of vibrocentrifuged concrete can be explained by achieving a denser packing of particles, in which nano-modifier particles act as crystallization centers. There is a redistribution of hydration processes and improvement of the structure formation of the binder-aggregate system, especially at the interfaces.

Thus, the action of the nano-modifier enhances the positive impact of the technological method of vibrocentrifugation, and all this leads to a notable improvement in the structure and compaction of the working layers of such concrete and to an increase in the strength characteristics, both differential and integral for elements made of such an improved variotropic concrete.

It can be seen from Figure 7, that the difference between the compressive strength of the inner layer of all types of vibrocentrifuged concretes with different dosages of GBFS differs slightly and varies from 34.2 MPa to 35.8 MPa. As for the compressive strength values for the middle layer of modified concretes, more significant changes are observed. Thus, the increase in compressive strength when replacing part of the cement with 10, 20, 30, 40, and 50% GBFS compared to the control composition was 7, 29, 32, 62, and 31%, respectively. The increases in compressive strength for the outer layer were 3, 3, 7, 7, and 1%, respectively.

After analyzing the dependence of the change in the difference between the compressive strength as a percentage for different layers, a tendency was established to reduce the difference between the middle and inner layers of vibrocentrifuged concrete modified with the addition of GBFS in the amount of 40% (Table 12).

The axial compressive strength of the inner layer of vibrocentrifuged concrete varied between 21.4 MPa and 22.3 MPa. As for the axial compressive strength values for the middle layer of modified concretes, the increases in compressive strength when replacing part of the cement with 10, 20, 30, 40, and 50% GBFS compared to the control composition were 10, 30, 39, 64, and 36%, respectively. The increases in axial compressive strength for the outer layer were 0, 4, 4, 12, and 3%, respectively.

The differences between the axial compressive strength as a percentage for different layers is shown in Table 13.

The flexural tensile strength of the inner layer of vibrocentrifuged concrete varied between 3.3 MPa and 3.4 MPa. As for the values of tensile strength in bending for the middle layer of modified concretes, the increments in tensile strength in bending when replacing part of the cement with 10, 20, 30, 40, and 50% GBFS compared to the control composition were 9, 30, 35, 61, and 39%, respectively. The increases in axial compressive strength for the outer layer were 1, 2, 3, 6, and 2%, respectively.

The differences between the tensile strength in bending as a percentage for different layers is shown in Table 14.

The axial tensile strength of the inner layer of vibrocentrifuged concrete varied between 2.0 MPa and 2.1 Mpa. As for the axial tensile strength values for the middle layer of modified concretes, the increases in axial tensile strength when replacing part of the cement with 10, 20, 30, 40, and 50% GBFS compared to the control composition were 6, 41, 44, 66, and 47%, respectively. The increases in axial tensile strength for the outer layer were 2, 4, 6, 8, and 4%, respectively.

The differences between the tensile strength in bending as a percentage for different layers is shown in Table 15.

The modulus of elasticity of the inner layer of vibrocentrifuged concrete varied between 25 MPa and 25.9 MPa. As for the values of the modulus of elasticity for the middle layer of modified concrete, the increase in the modulus of elasticity when replacing part of the cement with 10, 20, 30, 40, and 50% GBFS compared to the control composition was 7, 24, 28, 44, and 31%, respectively. The increases in axial tensile strength for the outer layer were 8, 8, 9, 14, and 5%, respectively.

The differences between the tensile strength in bending as a percentage for different layers is shown in Table 16.

Thus, from analyzing the experimental data, it was found that, at a dosage of between 0 and 40% of the cement substitution with GBFS, a stable increase in strength and deformation of integral characteristics by up to 22% is observed; however, at a dosage of more than 40%, a sharp decrease in these characteristics is observed. This is explained by the fact that, with a decrease in the proportion of cement, the ratio of reactive minerals of cement and slag does not provide an increase in the volume fraction of the products of the pozzolanic reaction, which contributes to an increase in the porosity of the hardened cement paste and, consequently, to a decrease in its strength.

Moreover, the modification of the GBFS makes it possible to increase the variotropic efficiency of the vibrocentrifuged concrete by reducing the differences between the strength characteristics of the middle and outer layers.

The values of the calculated coefficients of variotropic efficiency are presented in Table 17.

**Figure 12:**
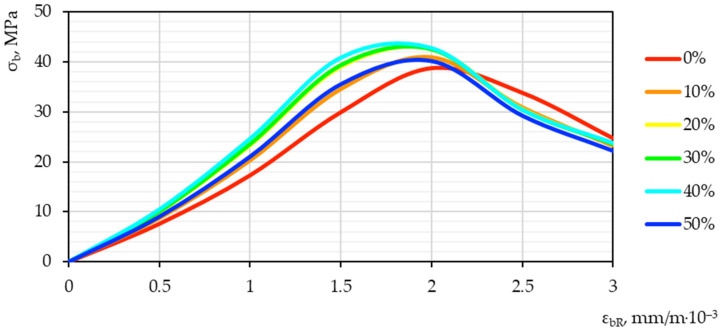
Diagram of “stress–strain” in compression for the integral characteristics of vibrocentrifuged concretes modified with the addition of GBFS.

**Figure 13:**
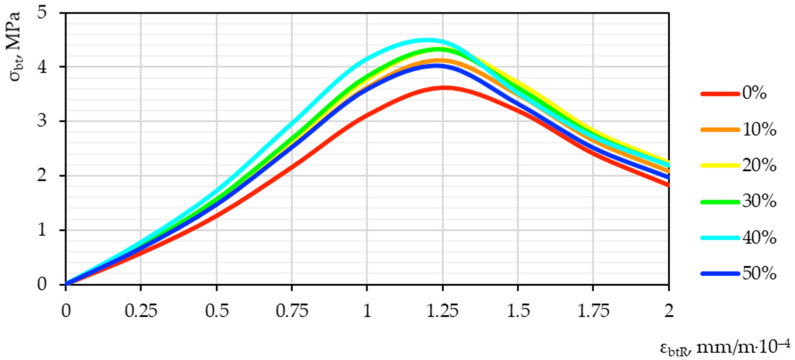
Diagram of “stress–strain” in tension for the integral characteristics of vibrocentrifuged concretes modified with the addition of GBFS.

Based on the test results, “stress–strain” diagrams “$\varepsilon_{bt}-\sigma_{bt}$” were constructed. Graphical dependences of “stress–strain” are presented in Figure 12 and Figure 13.

The optimal value, judging by the characteristic shift of the diagram peak up and to the left, is demonstrated by a sample with a nano-modifier dosage of 40%. Such a qualitative–quantitative picture illustrates the highest strength and lowest deformation characteristics of the resulting concrete. It is possible to explain the obtained effect by detecting an obvious optimum on the graph of the interdependence of the nano-modifier content and quality indicators. It is obvious that, with an increase in the amount of nano-modifier in the composition of concrete, there is also some preparation for a drop in performance after oversaturation with an excess amount of nano-modifier. Thus, after 40%, a certain decline begins, which is illustrated by the established dependence.

Obviously, concrete with a nano-modifier dosage of 40%, other things being equal, shows the greatest applicability in the practical industry. Thus, the highest strength characteristics allow it to provide the highest bearing capacity for structures based on it.

The deformability, which characterizes the shift of the peak of the diagram to the left, additionally shows that, other things being equal, such nano-modified concrete has the highest elastic modulus. The changes that occur can be explained by an increase in strength characteristics, that is, an increase in stress and a simultaneous decrease in strains indicators, since concrete acquires higher strength. Thus, the ultimate strains of such concrete are correspondingly reduced.

Of course, it would be expedient to apply concrete with these improved properties in practice in design and construction. 

### 3.3. Analysis of the Hardened Cement Paste Microstructure of Vibrocentrifuged Concrete Modified with the Addition of GBFS

This research of the microstructure was carried out to establish the relationship between the structure of the material and the strength characteristics of concrete. Samples were selected for the study, which were previously tested for compression.

**Figure 14:**
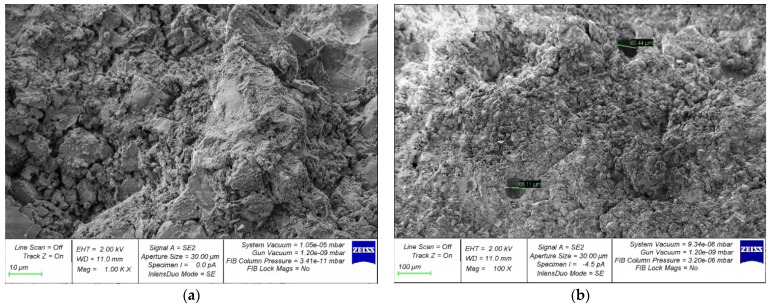
Microphotographs of the structure of hardened cement paste modified with the addition of GBFS in an amount of 10%: (**a**) 1000×; (**b**) 100×.

**Figure 15:**
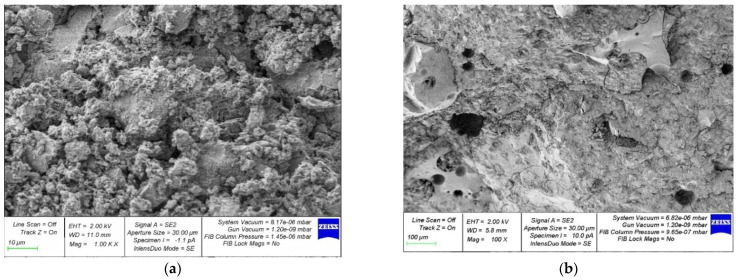
Microphotographs of the structure of hardened cement paste modified with the addition of GBFS in an amount of 20%: (**a**) 1000×; (**b**) 100×.

**Figure 16:**
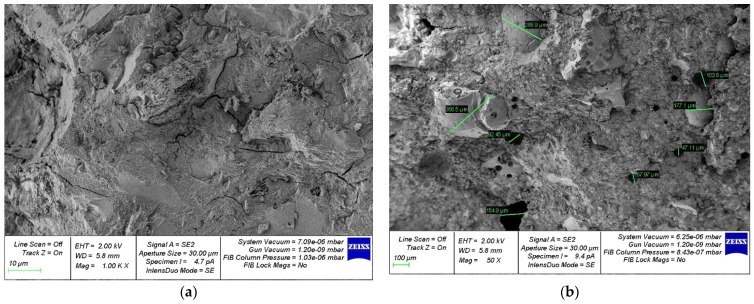
Microphotographs of the structure of hardened cement paste modified with the addition of GBFS in an amount of 30%: (**a**) 1000×; (**b**) 50×.

**Figure 17:**
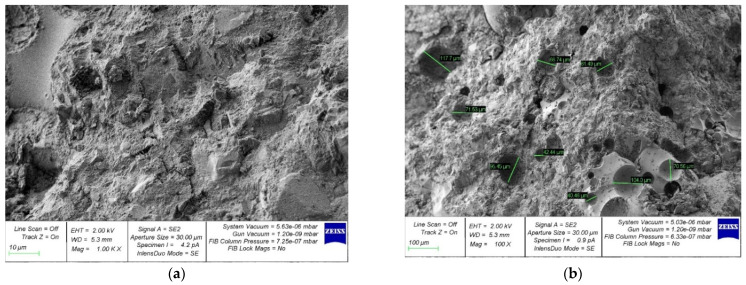
Microphotographs of the structure of hardened cement paste modified with the addition of GBFS in an amount of 40%: (**a**) 1000×; (**b**) 100×.

**Figure 18:**
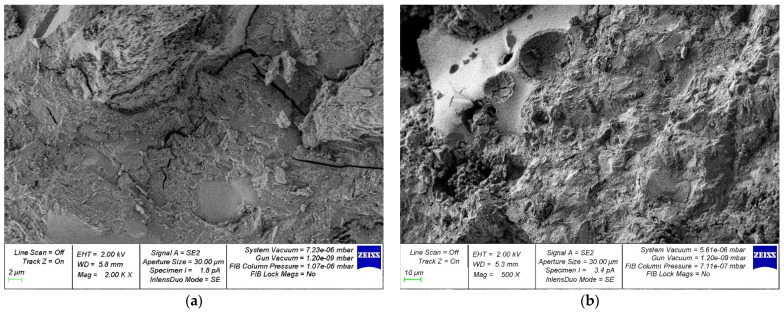
Microphotographs of the structure of hardened cement paste modified with the addition of GBFS in an amount of 50%: (**a**) 2000×; (**b**) 500×.

Figure 14, Figure 15, Figure 16, Figure 17 and Figure 18 show photos of the microstructures of hardened cement pastes with different dosages of GBFS.

Figure 14 shows that the microstructure of the samples of hardened cement paste modified with the addition of GBFS after destruction is loose; many micro cracks can be observed.

In Figure 15, Figure 16, Figure 17 and Figure 18, an almost similar picture to that in Figure 14 is observed. The obtained images indicate that the replacement of cement with a part of crushed GBFS provides an increase in the density of the microstructure of the hardened cement paste. Moreover, the introduction of the GBFS additive contributes to the formation of smaller cement hydration products with a size of less than five microns, which fill the main capillary pores. The addition of a nano-modifier, due to its fineness, allows hydration processes to occur more fully, becoming the centers of crystallization of these processes. This explains the effect obtained.

## 4. Discussion

The conducted studies show that the synergistic effect of a prescription factor (nano-modification), and technological impact (vibrocentrifugation) is expressed in a layer-by-layer increase in physical and mechanical characteristics and is explained by the formation of an improved variotropic structure. As a result of nano-modification, additional crystallization centers appear, and a denser packing of particles is formed in the most important layers—the middle and outer ones. Additional vibration during centrifugal compaction with rationally selected parameters results in a redistribution of the nature of variotropy with the values of the middle layer approaching the values of the outer layer. These structural transformations explain the achieved effect in the form of gains in both the integral and differential characteristics of new vibrocentrifuged nano-modified concretes.

For further analysis and comparison of the results obtained with the data obtained earlier in [44,45,46,47,48], three blocks can be conditionally distinguished: methodology, a structural component, and a resulting component. From the point of view of methodology, we have developed previously obtained theoretical concepts and applied dependencies arising from the influence of various prescription, technological, and other factors on the structure and properties of variotropic concretes. Earlier in this study, we established the fundamental possibilities and substantiated the recipe-technological feasibility of obtaining an improved variotropic structure due to the additional technological impact of adding a vibration component to centrifugal forces [44,45,46,47,48].

As is known from [43,44,45,46,47,48], vibrocentrifugation makes it possible to achieve an improved variotropic structure by obtaining an additional vibrational effect, influencing the rheological characteristics of the non-Newtonian fluid of the concrete mixture, which is in a rigid state due to the small amount of mixing water. This factor is a feature, and a distinctive feature, of the centrifugation mode, as well as vibrocentrifugation. At the same time, as is known, additional vibrational action leads to thixotropic liquefaction, which allows manufacturers to achieve better compactions of concrete and also, under certain conditions and with properly selected vibration modes, and hence vibrocentrifugation, to achieve better packing of particles at the macro level, thereby understanding and already possessing some knowledge about the achievability of improved structures due to the additional vibrational impact during vibrocentrifugation.

In [43], the authors also worked on the study of the bearing capacity of centrifugally compacted concrete with a variotropic structure. However, their main result considers the complex effect of the work of concrete and the material of the column pipe. Our result reflects the increase and variotropic efficiency of the concrete itself, which allows us to consider the achieved effects and the results obtained not only in the technological process, but also in the calculation and design of building structures. At the same time, unlike [43], we offer not pipe–concrete columns, but reinforced concrete columns.

Let us turn our attention to the possibilities of even greater improvement of this effect when exposed at the microlevel. At the microlevel, the picture is somewhat different. It is necessary to consider the specific features of the obtained variotropic structure of concrete mixtures of standard composition. Understanding these features and already possessing certain skills and specifics of the process, we can reliably say, after our study, that the impact with the help of nano-modifying additives, such as blast-furnace granulated slag, leads to an even greater effect, including the ability to achieve a synergistic effect in the formation of improved variotropic structures.

Previously, in [47], we obtained certain results that related to the technological and prescription advantages of the nano-modification of vibrocentrifuged concretes using microsilica. Important results were achieved, which led to an improvement in strength properties by 30% and a change in deformation properties by 34%, and the economic efficiency of the proposed methods was also substantiated. At the same time, in this work, we have developed the already obtained primary theory of nano-modification of variotropic structures of concrete, as well as expanded applied concepts and practical dependencies for an even deeper study of the process of improving concrete with the help of nano-modifiers; we have also provided specific recommendations for the construction industry in terms of the use of nano-modifier blast furnace granulated slag. Thus, from the point of view of the methodology of the structure and results, significant progress has been made in the study of the nano-modification of variotropic structures of concrete.

The nanomodification of the variotropic structure with finely ground blast-furnace granulated slag has shown its high efficiency in terms of results at the macro level. This was confirmed by the results of the laboratory tests of the prototypes. Let us consider the fundamental picture that occurs at the microlevel during the formation of structure and the formation of the properties of such concretes. By analogy with the previously established dependencies [47,55], the nanomodifier allows, with optimal recipe-technological parameters, the creation of additional crystallization centers, which in turn lead to improvements in all processes, from hydration to the formation of a denser concrete structure with more dense packing of particles at the microlevel. Add to this the impact of particles at the macro level, which consists of the use of vibrocentrifugation with rational parameters. The rational parameters of vibrocentrifugation make it possible to achieve not only an improvement in the integral properties of concrete, that is, the general properties averaged over the annular variotropic section, but also to achieve the most advantageous differentiation of properties. At the same time, the differentiation of properties in most of the working layers of the section reaches the highest values, and the layers located closer to the inner non-working zone do not receive additional crystallization centers, and, thus, the resources spent on obtaining the most perfect structures are not spent. Thus, the energy and economic efficiency of the achieved result is confirmed. Energy efficiency is achieved due to the lowest labor costs and equipment operation aimed at the formation of an improved variotropic structure. Economic efficiency is achieved by replacing some of the expensive components with blast-furnace granulated slag, which, in fact, is a waste product. Thus, at the macro- and microlevels, both on the recipe and on the technological example, the effectiveness of the ideas proposed by the authors is confirmed.

## 5. Conclusions

When summarizing the results of the theoretical and experimental research conducted in this study, the following conclusions can be drawn. Firstly, we have developed previously obtained theoretical and practical results concerning the developed concept of the nano-modification of variotropic concrete structures. The purpose of nanomodification is to obtain concrete with improved characteristics and greater operational reliability.

The most desirable prescription–technological factors were experimentally derived and confirmed at the microlevel using structural analysis. Applied and mathematical dependencies among the composition, macrostructure, microstructure, and final properties of the vibrocentrifuged concretes nano-modified by blast-furnace granulated slag were determined. Empirical relationships were identified to express the variation of some mechanical parameters and identify the relationship between them and the composition of the mixture.

The optimal dosage of granulated blast-furnace slag for the nano-modification of variotropic vibrocentrifuged concretes was determined, which is 40%.

The influence of the optimal dosage of blast-furnace granulated slag on the properties of nano-modified vibrocentrifuged variotropic concretes was revealed, which was reflected in the quantitative ratio as an increase in density of 3%, strength indicators—from 16% to 27% (depending on the type of strength), and elasticity modulus—of 23%.

Further prospects for the study were determined, which consist of continuing research on the effect of nano-modifying additives on variotropic vibrocentrifuged concretes, in particular, using even more finely dispersed materials brought to the nano-level: nano-silica, nano-slag, and nano-fly ash, as well as nanosized ash, rice husks, and rice straw.

To cover the most problematic areas of the accumulation of various wastes, both industrial and agricultural waste, as well as waste from the fuel and energy complex (fuel industry (coal, gas, oil, shale, peat) and electric power industry), are proposed to be used in concrete technology, and, in view of the proven effectiveness of the use of such additives in concretes of variotropic structures, this direction is proposed to be researched as a priority.

## Figures and Tables

**Table 1:** The results of determination of strength properties [17].

Holding Time, Days	Holding Temperature, °C			
	22		65	
	Compressive Strength, MPa	Tensile Strength in Bending, MPa	Compressive Strength, MPa	Tensile Strength in Bending, MPa
7	31.9 ± 1.6	6.3 ± 0.4	30.0 ± 1.9	5.3 ± 0.3
28	63.5 ± 1.2	13.5 ± 1.2	53.3 ± 0.3	11.4 ± 0.8

**Table 2:** Mineralogical composition of Portland cement.

Mineral	Content, %
C_3_S	67
C_2_S	15
C_3_A	7
C_4_AF	11

**Table 3:** Grain composition and modulus of sand size.

Residues on Sieves, %	Sieves Diameter, mm						Size Modulus
	2.5	1.25	0.63	0.315	0.16	<0.16	
Partial	1.28	1.28	10.51	45.04	39.74	2.15	1.73
Full	1.28	2.56	13.07	58.11	97.85		

**Table 4:** Characteristics of crushed granite.

Indicator Title	Value
Fraction size	5–10
Bulk density, kg/m^3^	1487
True density, g/cm^3^	2.65
Crushability, % by weight	11.6
The content of grains of lamellar (flaky) and acicular forms, % by weight	9.1
Voidness, %	44

**Table 5:** Chemical composition of GBFS.

Material	Content, wt. %
SiO_2_	26.56
CaO	56.35
MgO	6.42
Al_2_O_3_	5.92
Na_2_O	1.07
K_2_O	0.29
TiO_2_	2.37
MnO	0.18
S	0.32
Fe_2_O_3_	0.33
SrO	0.14
P_2_O_5_	<0.01
ZrO_2_	0.03
Co_3_O_4_, V_2_O_5_, Cr_2_O_3_, Nd_2_O_3_, WO_3_, Ta_2_O_5_, Nb_2_O_5_, Sc_2_O_3_, Y_2_O_3_, BaO, CuO	<0.01

**Table 6:** List of formulas for assessing the variotropic efficiency of vibrocentrifuged concretes.

Calculation of Average Values of Indicators According to Experimental Data of Differential Characteristics	Calculation of Coefficients of Variotropic Efficiency According to Experimental Data
$\mathrm{Compressive}\;\mathrm{strength}\;\bar{R_{b,cub}}=\frac{\sum_{i=1}^{n}R_{b,cub\;i}}{n}$	$K_{R_{b,cub}}=\left|\frac{\bar{R_{b,cub}}-R_{b,cub}}{R_{b,cub}}\right|\times 100,\;\%$
$\mathrm{Axial}\;\mathrm{compressive}\;\mathrm{strength}\;\bar{R_{b}}=\frac{\sum_{i=1}^{n}R_{b\;i}}{n}$	$K_{R_{b}}=\left|\frac{\bar{R_{b}}-R_{b}}{R_{b}}\right|\times 100,\;\%$
$\mathrm{Bending}\;\mathrm{tensile}\;\mathrm{strength}\;\bar{R_{btb}}=\frac{\sum_{i=1}^{n}R_{btb\;i}}{n}$	$K_{R_{btb}}=\left|\frac{\bar{R_{btb}}-R_{btb}}{R_{btb}}\right|\times 100,\;\%$
$\mathrm{Axial}\;\mathrm{tensile}\;\mathrm{strength}\;\bar{R_{bt}}=\frac{\sum_{i=1}^{n}R_{bt\;i}}{n}$	$K_{R_{bt}}=\left|\frac{\bar{R_{bt}}-R_{bt}}{R_{bt}}\right|\times 100,\;\%$
$\mathrm{Elastic}\;\mathrm{modulus}\;\bar{E_{b}}=\frac{\sum_{i=1}^{n}E_{b\;i}}{n}$	$K_{E_{b}}=\left|\frac{\bar{E_{b}}-E_{b}}{E_{b}}\right|\times 100,\;\%$

Note: *i*—layer number; *n*—number of layers.

**Table 7:** Parameters of the composition of the concrete mixture.

Indicator	W/C	Absolute Volume of Cement Paste, L	Absolute Volume of Aggregates, L, at a Ratio r = S/CS = 0.4	Weight of Cement in the Mix, kg/m^3^	Weight of Crash Stone in the Mix, kg/m^3^	Weight of Sand in the Mix, kg/m^3^
Indicator value	0.38	319	1805	400	1290	515

**Table 8:** Quality indicators of blast-furnace slag.

Indicator	Value
Basicity modulus	1.93
Activity index	4.81
Quality factor	2.12
Grade according to GOST 3476	1

**Table 9:** Granulometric characteristics of GBFS.

Particle Size, %	Fraction Content, % at Grinding Time, Min			
	10	20	30	40
up to 25 µm	28.6	42.6	76.3	73.1
up to 50 µm	18.5	24.5	16.4	11.7
up to 100 µm	39.2	27.0	4.8	9.7
over 100 µm	13.7	5.9	2.5	5.5

**Table 10:** Test results of sample cubes.

Characteristics	Grinding Time GBFS, Min			
	10	20	30	40
Compressive strength, MPa	56.7 ± 3.2	58.5 ± 3.5	64.8 ± 3.7	62.9 ± 3.8

**Table 11:** Experimental data of the effect of the addition of GBFS on the integral characteristics of modified vibrocentrifuged concrete.

Concrete Characteristics	Dosage of Mineral Additive, wt. % Replacement of Cement					
	0	10	20	30	40	50
ρ, kg/m^3^	2412 ± 53	2419 ± 56	2445 ± 54	2468 ± 52	2485 ± 50	2461 ± 59
R_b,cub_, MPa	51.9 ± 3.1	55.9 ± 3.4	59.7 ± 3.7	60.5 ± 3.6	62.9 ± 3.4	57.3 ± 3.3
R_b_, MPa	38.8 ± 2.2	40.8 ± 2.5	43.9 ± 2.9	44.0 ± 2.8	45.0 ± 2.5	40.7 ± 2.6
R_btb_, MPa	6.2 ± 0.4	6.6 ± 0.6	7.1 ± 0.5	7.3 ± 0.4	7.9 ± 0.4	7.2 ± 0.6
R_bt_, MPa	3.6 ± 0.2	4.1 ± 0.3	4.3 ± 0.3	4.3 ± 0.2	4.5 ± 0.2	4.0 ± 0.3
E_b_, GPa	33.7 ± 2.0	35.7 ± 2.2	38.5 ± 2.3	38.7 ± 2.1	41.5 ± 2.2	35.1 ± 2.2

**Table 12:** Difference between compressive strength for different layers of vibro-centrifuged concrete.

Inner and Middle Layer Distinction						Inner and Outer Layer Distinction						Middle and Outer Layer Distinction					
Dosage of GBFS, wt. % Replacement of Cement																	
**0**	**10**	**20**	**30**	**40**	**50**	**0**	**10**	**20**	**30**	**40**	**50**	**0**	**10**	**20**	**30**	**40**	**50**
28	32	44	46	55	44	53	54	55	56	56	53	35	32	19	19	2	15

**Table 13:** Differences between axial compressive strength for different layers of vibrocentrifuged concrete.

Inner and Middle Layer Distinction						Inner and Outer Layer Distinction						Middle and Outer Layer Distinction					
Dosage of GBFS, wt. % Replacement of Cement																	
**0**	**10**	**20**	**30**	**40**	**50**	**0**	**10**	**20**	**30**	**40**	**50**	**0**	**10**	**20**	**30**	**40**	**50**
39	43	53	58	62	54	62	60	63	65	66	61	37	31	22	17	9	17

**Table 14:** Differences between tensile strength in bending for different layers of vibrocentrifuged concrete.

Inner and Middle Layer Distinction						Inner and Outer Layer Distinction						Middle and Outer Layer Distinction					
Dosage of GBFS, wt. % Replacement of Cement																	
**0**	**10**	**20**	**30**	**40**	**50**	**0**	**10**	**20**	**30**	**40**	**50**	**0**	**10**	**20**	**30**	**40**	**50**
40	45	55	57	64	57	62	62	64	64	65	63	36	31	19	16	2	13

**Table 15:** Differences between axial tensile strength for different layers of vibro-centrifuged concrete.

Inner and Middle Layer Distinction						Inner and Outer Layer Distinction						Middle and Outer Layer Distinction					
Dosage of GBFS, wt. % Replacement of Cement																	
**0**	**10**	**20**	**30**	**40**	**50**	**0**	**10**	**20**	**30**	**40**	**50**	**0**	**10**	**20**	**30**	**40**	**50**
38	38	56	54	62	55	62	60	63	62	64	61	38	36	17	16	5	13

**Table 16:** Differences between the modulus of elasticity for different layers of vibrocentrifuged concrete.

Inner and Middle Layer Distinction						Inner and Outer Layer Distinction						Middle and Outer Layer Distinction					
Dosage of GBFS, wt. % Replacement of Cement																	
**0**	**10**	**20**	**30**	**40**	**50**	**0**	**10**	**20**	**30**	**40**	**50**	**0**	**10**	**20**	**30**	**40**	**50**
18	23	33	37	42	37	38	42	42	44	45	41	24	25	13	12	4	6

**Table 17:** Values of coefficients of variotropic efficiency.

Variotropic Efficiency Coefficient	Dosage of GBFS, wt. % Replacement of Cement					
	0	10	20	30	40	50
$K_{R_{b,cub}}$	1.7	1.6	2.2	1.2	3.0	1.8
$K_{R_{b}}$	2.9	3.9	4.4	3.1	5.9	5.0
$K_{R_{btb}}$	3.2	6.1	7.0	7.3	7.6	5.6
$K_{R_{bt}}$	3.7	4.4	7.8	5.4	8.4	1.4
$K_{E_{b}}$	3.1	3.2	5.4	5.4	5.6	4.6

## Data Availability

The study did not report any data.
